# Consistent Optimization of Blast Furnace Ironmaking Process Based on Controllability Assurance Soft Sensor Modeling

**DOI:** 10.3390/s22124526

**Published:** 2022-06-15

**Authors:** Junfang Li, Chunjie Yang, Chong Yang

**Affiliations:** State Key Laboratory of Industrial Control Technology, College of Control Science and Engineering, Zhejiang University, Hangzhou 310027, China; yooo-li@zju.edu.cn (J.L.); yangchong2020@zju.edu.cn (C.Y.)

**Keywords:** blast furnace, soft sensor, mixed density networks, consistency optimization

## Abstract

The blast furnace ironmaking process is the core of steel manufacturing, and the optimization of this process can bring enormous economic and environmental benefits. However, previous data-driven optimization methods neglect the uncontrollability of part of the variables in the predictive modeling process, which brings great uncertainty to the optimization results and adversely affects the optimization effect. To address this problem, a consistency optimization framework based on controllability assurance soft sensor modeling is proposed. The method achieves the information extraction of uncontrollable variables in a process-supervised way, and improves the posterior distribution prediction accuracy. The method also proposes an integrated self-encoder regression module, which uses the regression to guide the encoding, realize the construction of latent features, and further improve the prediction accuracy of the model. Integrating the prediction module and the multi-objective gray wolf optimizer, the proposed model achieves the optimization of the blast furnace ironmaking process with only controllable variables as prediction model inputs while being capable of giving uncertainty estimates of the solutions. Empirical data validated the optimization model and demonstrated the effectiveness of the proposed algorithm.

## 1. Introduction

Steel manufacturing is an essential foundation of the world industry. The energy consumption of this process accounts for about 10% of the total global energy consumption [1], while its carbon dioxide emissions also account for a significant share. The blast furnace ironmaking process as the core of steel manufacturing is the most prominent carbon dioxide emitting process, and its energy consumption accounts for 65% of the steel manufacturing process [2]. The quality of the product in this process also directly impact the energy consumption and emissions of subsequent processes. Thus, a minor enhancement of the blast furnace iron making process, such as reducing energy consumption, emissions or improving product quality, can bring significant economic and environmental benefits.

**Figure 1:**
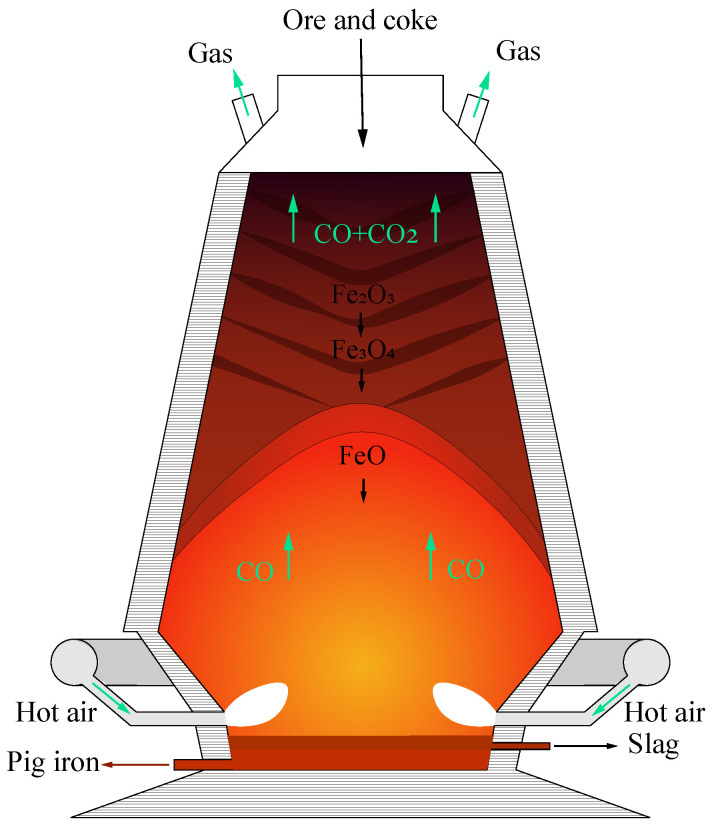
Blast furnace ironmaking process.

The blast furnace iron making process is complicated. The process is shown in Figure 1. Firstly, raw materials such as iron ore and coke are loaded from the top of the furnace, and complicated physicochemical reactions take place in a high temperature and high pressure environment to finally produce molten iron. Due to the complexity of the process, existing blast furnace control and optimization is simply performed through workers’ experience. This leads to additional waste and makes it hard to improve the product quality.

In fact, studies on the optimization of this process have been conducted for a long time. Ref. [3] examines the chemical activation of blast-furnace slag pastes with alkaline solutions by means of various characterization techniques. Ref. [4] studied coke behavior in an operating blast furnace with the main emphasis being on the role of its inherent mineral matter. However, due to the complexity of the industrial process, it is challenging to build the mechanistic models, which makes it hard for the the research methods to work. It was not until data-driven metaheuristic algorithms were developed for the optimization of industrial processes. In order to obtain the best material surface distribution, Ref. [5] first uses a kernel extreme learning machine to build a prediction model for the relevant indexes, and then solves the optimal solution for the decision variables related to the material surface shape by a heuristic algorithm. Ref. [6] combined machine learning methods and particle swarm algorithms to achieve optimization of industrial propane dehydrogenation processes. Ref. [7] proposed a two-stage optimization algorithm that first implemented a Mixed Data Sampling regression model based on the conditional weighting of the mixed flow, and then used a population-adaptive genetic algorithm to achieve energy consumption reduction in the paper-making process. Based on subtractive clustering and adaptive neuro-fuzzy inference, Ref. [8] established a high-precision prediction model of fiber quality and process energy consumption in the paper refining process, based on which the guidance of medium density fiberboard production process was realized by using the simulated annealing method. Finally, the fiber quality is improved while the process energy consumption is reduced.

It can be seen that a common prerequisite for the working of the above research methods is the ability to build accurate data-driven predictive models. In fact, there are plenty of studies on soft sensors of industrial processes [9,10,11]. Ref. [12] implemented industrial soft sensor modeling using support vector machines (SVM), while optimal SVM model selection was achieved using a Bayesian evidence framework. Accurate prediction and variation tracking of the freezing point of light diesel fuel was achieved. Ref. [13] proposed a semi-supervised gaussian mixture model (GMM) regression model utilizing both labeled and unlabeled samples to achieve a soft measurement of the process based on expectation maximization. Ref. [14] designed a dynamic convolutional neural networks (CNN)strategy for learning hierarchical local nonlinear dynamic features. The method considers both temporal and spatial features of the process and establishes a high-precision soft measurement model. Ref. [15] proposed a dynamic feature extraction model, using the encoder–decoder structure to achieve dynamic feature extraction, and a dynamic feature smoothing method using attention weights to achieve denoising and reduce model overfitting, which greatly improves the robustness and prediction accuracy of the model.

There is also a lot of soft sensor research in the blast furnace ironmaking process. Ref. [16], inspired by the digital twin, combined the CFD model and the support vector machine (SVM) model to achieve the real-time estimation of raceway depth of the blast furnace. Ref. [17] built a multistep prediction model, called a denoising spatial-temporal encoder–decoder, for the prediction of burn-through point (BTP) in advance. Ref. [18] proposed an adaptive stacked polymorphic model to realize the online measurement of the silicon content of molten iron.

The essence of these soft sensor methods is to extract the relevant information from the process variables that are used as inputs to the model to achieve the prediction of quality variables. When the process variables are used only for predicting quality indexes, there is no need to be concerned about whether the variables are controllable or not. However, when the prediction model is used as the fitness function of the heuristic algorithm, the controllability of the inputs of the model matters a lot. The controllability is illustrated here by means of instances. In general, the controllability of the model inputs can be guaranteed in two cases. First, all the input variables of the model are controllable variables. Second, the input variables of the model are partly controlled variables and partly not, but the uncontrolled variables are independent from the controlled ones, so that the uncontrolled variables can be fixed only to assist in predicting the quality variables, and adjust the controlled variables without affecting the uncontrolled ones during optimization.

In fact, to ensure the accuracy of the prediction model, the controllability of the input variables of the model cannot be guaranteed in some industrial processes. For example, in the blast furnace ironmaking process, the prediction of iron quality-related indicators requires the collection of as many relevant variables as possible, including raw materials grade, operating variables, and detection variables. The detection variables data are critical process information measured by the sensors and can provide valid information to predict the quality indicators. However, they are not independent of the operation variables. As the operating variables are adjusted, the detection variables will change as well. There is a lack of research related to properly addressing this issue.

To address this problem, this paper proposes an optimization framework based on process-supervised distribution regression aided by uncontrollable variables. The optimization method is able to extract information of the detection variables while keeping them from being used as input in the prediction stage. It aims to improve the prediction accuracy of the distribution of quality indicators as much as possible under the conditions of available information, and thus to obtain reliable optimization results with uncertainty estimates. The main contributions of the paper include:

1. A controllability assurance modeling method is proposed. Uncontrollable variables are used as supervised information to assist feature construction in the training phase, enabling information extraction without being used as input in the prediction phase and improving the prediction accuracy of the distribution of quality variables.
2. A semi-supervised autoencoder regression method is proposed. The method combines the autoencoder and regression methods into an end-to-end integrated model to achieve feature extraction and improve model prediction accuracy.
3. A consistency optimization framework is proposed. Combining the above feature extraction, the distribution regression method, and the multi-objective gray wolf algorithm, the consistency optimization of the operating parameters and detection variables is achieved.

The rest of this paper is organized as follows: Section 2 presents the background of data-driven metaheuristics. Section 3 consists of the description of the optimization problem and explanation of the proposed method. Section 4 illustrates the experiments and results corresponding to the proposed techniques. Ultimately, Section 5 presents the conclusions.

## 2. Data-Driven Metaheuristics

**Figure 2:**
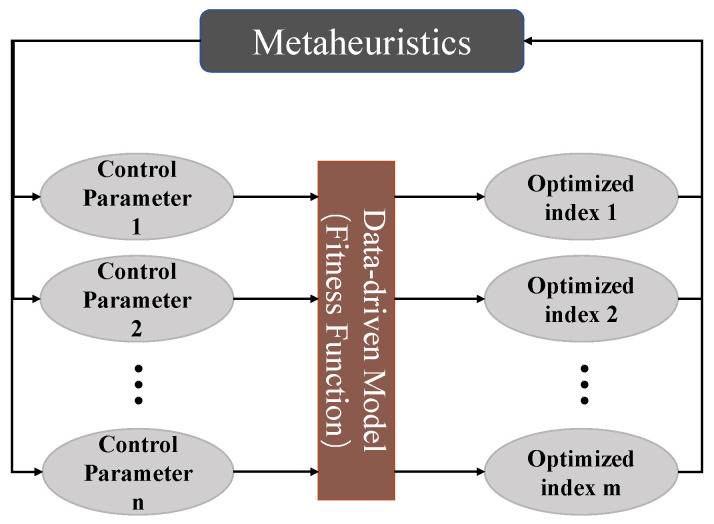
Illustration of the data-driven metaheuristic algorithm.

Data-driven meta-heuristics combining machine learning with meta-heuristic algorithms to find high performance outcomes in the process industry have achieved great success [19,20,21]. The fitness function as the primary component of the Metaheuristic algorithm is the basis of optimization [22]. However, it is challenging to build an accurate mathematic model in some complex processes. In contrast, data-driven methods do not have to focus on the details of physical and chemical reactions of the industrial process but only use historical data to achieve modeling, which has excellent potential for modeling the complex process. Thus, the prediction model built by machine learning can be used as the fitness function of the heuristic algorithm to evaluate the quality of the search agents, as shown in Figure 2.

In the metaheuristic algorithm, the inputs of the fitness function are usually controllable parameters, and the outputs are the optimized values of the corresponding achievable optimal indicators. The optimal combination of operational parameters is searched to achieve production index optimization.

It should be noted that the gray wolf optimizer (GWO) algorithm is adopted as the basic optimizer of the proposed algorithm because of its low computational complexity and high search efficiency.

## 3. Methodology

### 3.1. Optimization Problem Description

**Figure 3:**
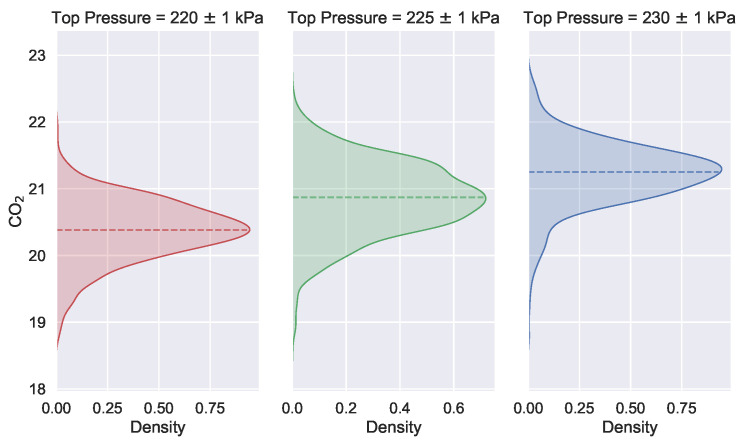
Variation of the posterior distribution of the detection variables with the change of operational variable. The horizontal axis is the probability density value of the distribution of the carbon dioxide. The vertical axis shows the value of the volume fraction of the carbon dioxide flow at the top of the furnace.

As mentioned earlier, the key of data-driven heuristic algorithms is to build an accurate prediction model. The inputs to the fitness function need to be of controllability. However, the information provided by the detection variables is extremely critical when using a data-driven approach for predictive modeling of process indicators in the blast furnace ironmaking process. The detection variables are always uncontrollable and influenced by the operating variables. As shown in Figure 3, the graph indicates that the distribution of the carbon dioxide emission flow rate of the production process changes with the variation of the top pressure of the furnace. It can be seen that, as the furnace top pressure increases, the carbon dioxide emissions increase as well.

In fact, some operating variables such as top temperature and top pressure can be controlled during the ironmaking production process. However, the difficulty of control and the requirement for the smooth operation of the process also make people reluctant to adjust them. Therefore, these variables are also considered as uncontrollable detection variables in the optimization process in this paper.

**Figure 4:**
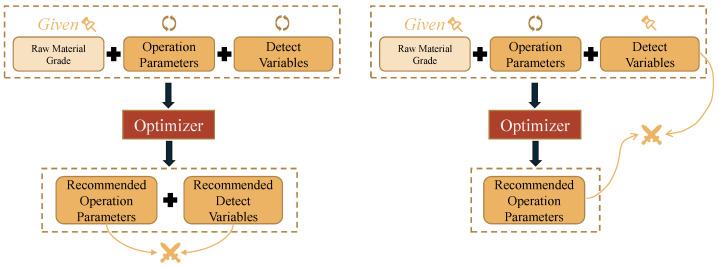
Illustration of the inconsistency of traditional methods’ optimization results.

However, existing optimization methods have disregarded this uncontrollability. As shown in Figure 4, the traditional optimization method on the left can perform searching independently in the feasible domain of both the operating parameters and the detection variables, given the raw material grade as the environment variable. The final optimized solution is the recommended operating parameters and detection variables; however, the fact is that the detection variable is not controllable. The actual controllable parameters are only the operating parameters, so the process indexes corresponding to the recommended operating parameters may not be optimal.

In the next case, considering that the detection variables are not controllable, they are fixed as the raw materials grade. The search is performed only in the feasible domain of the operating parameters. The final optimized solution is the recommended operating parameters. There is still a problem that the recommended operating parameters may not be consistent with the fixed detection variables.

### 3.2. Controllability Assurance Modelling Method

The detection variables, etc., are not controllable but the key variables for production indices’ prediction. The first thought is trying to regress the detection variables like other expert constructed features such as Permeability Index, Pressure Differential, etc. and then use them to make predictions.

**Figure 5:**
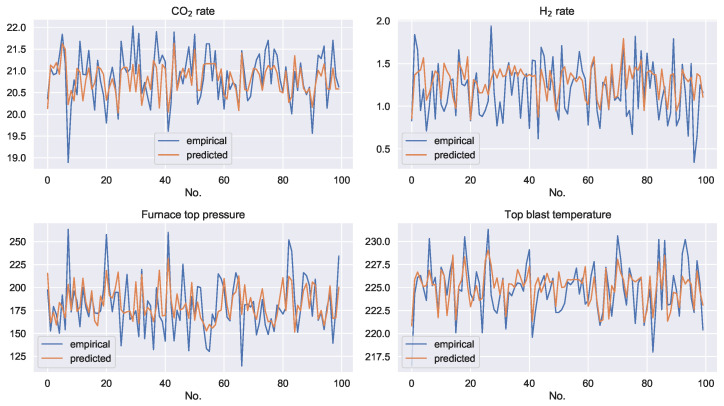
Regression results for the uncontrollable variables. The horizontal axis of each subplot indicates the numbers of the samples. The vertical axis indicates the value of each variable.

The prediction results of the support vector machine regression model with controllable variables as inputs for the four uncontrollable variables are shown in Figure 5. The blue line is the empirical data and the orange line is the predicted data. It can be seen that, although the controllable variables contain certain information from the uncontrollable variables, the uncontrollable variables still have independent information components. The controllable variables are not able to regress the exact values of the controllable variables.

Therefore, the proposed method aims to achieve the extraction of physical features related to the detection variables from the operational variables by using the historical data of the detection variables, and to estimate the independent components of the detection variables.

Since the regression of exact values cannot be achieved, it is desired to obtain the posterior distribution of the uncontrollable variables for given controllable variables. It means that the output of the desired model is no longer the predicted values of the variables but the parameters of the variable distribution model. We can implement this idea using a mixture density network (MDN) [23]. Any probability distribution can be expressed as a linear combination of several Gaussian components [24]. The original MDN model combines the Gaussian mixture model and the neural network. In addition, the output layer of the network is the parameters of the Gaussian mixture model. The conditional probability distribution of the detection variables can be represented by a mixture of multiple Gaussian components.

**Figure 6:**
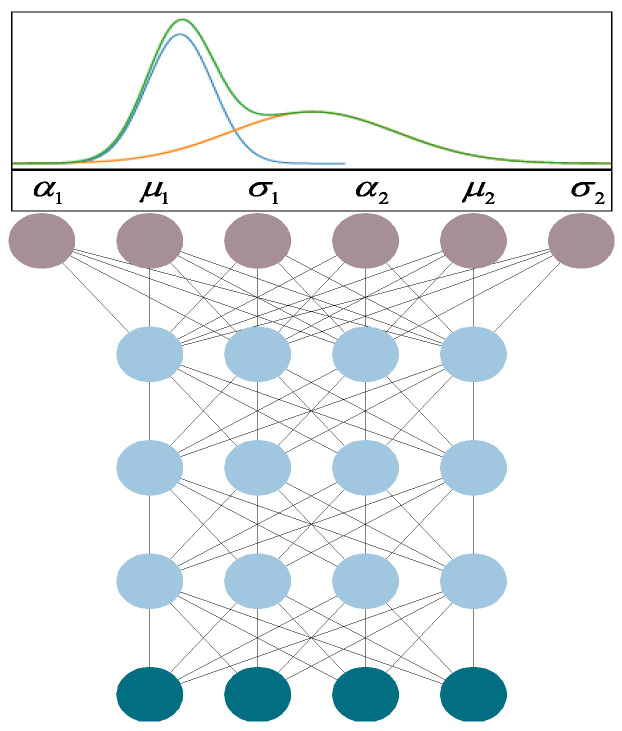
Mixture density networks structure.

Figure 6 illustrates the structure of the original MDN model, with the dark green neurons representing the input layers of the model and the three hidden layers in the middle. The output layer here outputs the parameters of the model of a mixture of two Gaussian components. The blue curve represents the first Gaussian component, the orange curve describes the second Gaussian component, and the green curve represents the posterior probability distribution after mixing the two Gaussian components. For any operational parameter *x*, the well-trained mixture density network is able to give the posterior probability distribution p(y|x) of the detection variable *y* as:

(1) $$p\left(y|x\right)=\sum_{i=1}^{m}\alpha_{i}\left(x\right)N_{i}\left(y|\mu_{i}\left(x\right),\sigma_{i}^{2}\left(x\right)\right)$$

where *m* is the number of Gaussian components, α is the weight coefficient of the Gaussian component; and $\mu_{i}$ and $\sigma_{i}$ represent the mean and variance of the corresponding *i*-th Gaussian component. It should be noted that the sum of the Gaussian component weight coefficients should be equal to 1:

(2) $$\sum_{i=1}^{m}\alpha_{i}\left(x\right)=1$$

Therefore, the activation function of the network corresponding to the output is the softmax function:

(3) $$\alpha_{i}\left(x\right)=\frac{\exp\left(e_{i}^{\alpha }\left(x\right)\right)}{\sum_{j=1}^{m}\exp\left(e_{j}^{\alpha }\left(x\right)\right)}$$

where $e_{i}^{\alpha }$ represents the un-normalized *i*-th Gaussian component weight coefficients of the model output:

(4) $$\sigma_{i}=\exp\left(e_{i}^{\sigma }\right)$$

(5) $$\mu_{i}=e_{i}^{\mu }$$

where $e_{i}^{\mu }$ and $e_{i}^{\sigma }$ are the neural network outputs corresponding to the mean and variance of the *i*-th Gaussian component.

The cost function of the MDN model is the negative loglikelihood of the observation *y* given its input *x*. Here, we can formulate the loss function as:

(6) $$\log L\left(y|x\right)=-\log\left(\sum_{i=1}^{m}\alpha_{i}\left(x\right)N_{i}\left(y|\mu_{i}\left(x\right),\sigma_{i}^{2}\left(x\right)\right)\right)$$

It should be noted that, when it comes to solving for the joint posterior distribution of multiple variables, the σ in the above equation represents the elements in the covariance matrix of the variables. This means that, when the variables are not independent, the model also needs to predict the covariances among the variables. When the variables are not independent, the number of model output units proliferates, harming model training and accuracy. Accordingly, the proposed method performs principal component analysis (PCA) on uncontrollable variables to remove variable correlations. It should be noted that the PCA procedure does not reduce the dimensionality of the variables. It is only used to reduce the model structure’s complexity and improve the distribution prediction accuracy.

**Figure 7:**
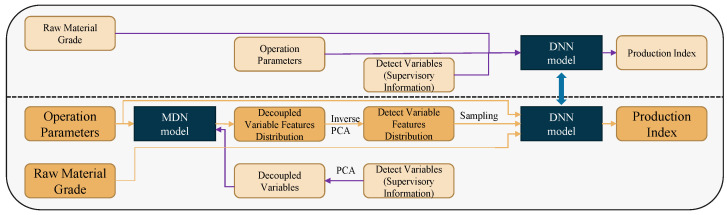
Illustration of a controllability assurance modeling method.

The main idea of the controllability assurance modeling (CAM) method is shown in Figure 7. As shown above the dashed line in the figure, a deep neural network (DNN) is firstly trained with raw materials grade parameters, operational parameters, and detection variables as model inputs and production index as output. The detection variables cannot be used in the actual prediction process, so it is required to predict the distribution of the corresponding features of the detection variable using the operational parameters. Then, the sampling is performed under the distribution, and the sampled data are obtained to replace the original detection variables data.

To predict the distribution of the features corresponding to the detection variables, the MDN model is trained in the training phase with the historical detection variable data as the supervised information and the operational parameters as the inputs.

It can be seen that, to reduce the complexity of the MDN model structure, PCA decoupling is performed on the detection variables data. In addition, PCA inversion is performed on the sampled feature data. The elements represented by the light-colored boxes and the purple flow lines in the figure are only involved in the model training phase. The dark orange boxes and the orange flow lines in the figure show the prediction process. In the prediction process, the operational variables are used as inputs of the MDN model to obtain the posterior distribution. Sampling is performed under the distribution to obtain the sampled data, and multiple feature data corresponding to detection variables are obtained after inverse PCA transformation. Operational parameters and raw materials grade data can be concatenated to predict a production index value using the well-trained DNN model for any of the sampled feature data. In this way, multiple production index predictions can be obtained, and the mean and variance of the predictions can be statistically calculated to measure the expectation and uncertainty of the prediction results.

### 3.3. Beta Self-Encoding Regression Model

Inspired by feature extraction under the supervision of detection variables, the beta self-encoder regression (βSER) method is proposed, which can generate deep features under the self-supervision of operational parameters and use the extracted features to improve the prediction accuracy of production indexes.

It should be noted that, in order to make sure that the generated features can effectively improve the model prediction accuracy, different from the general regression method after autoencoder feature extraction, the autoencoder and regression methods are combined into an end-to-end integrated model. In this case, the encoder needs to consider both reconstruction error and regression error. The loss function of the model is shown below:

(7) $$loss=\frac{1}{N}\sum_{i}^{N}{∥y_{i}-{\hat{y}}_{i}∥}^{2}+\beta *\frac{1}{N}\sum_{i}^{N}{∥x_{i}-{\tilde{x}}_{i}∥}^{2}$$

where *N* represents the number of samples, $x_{i}$, ${\tilde{x}}_{i}$ represents the *i*-th operational variables input sample and reconstructed sample, respectively. In addition, ${\hat{y}}_{i}$ and $y_{i}$ represent the *i*-th predicted and true value, respectively. β is a hyperparameter to balance the effect of reconstruction error and prediction error on the encoder. It can be seen from the loss function that the reconstruction error and the prediction error are interacting with each other. The model prediction can be hampered when the weight of reconstruction error in the loss function is too big. When the weight of reconstruction error in the loss function is too small, the validity of the encoder features cannot be guaranteed. In addition, an appropriate weight can help the model construct valuable features and improve the prediction accuracy of the model.

**Figure 8:**
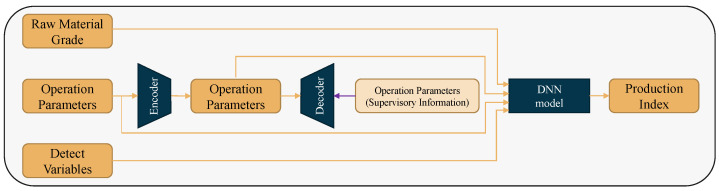
Schematic diagram of the self-encoding regression method.

The main idea of the proposed method is shown in Figure 8. The self-encoder accomplishes deep feature extraction while reconstructing operational parameters. The deep features are concatenated with the raw materials grade parameters, operating parameters and detection variables to predict the production index.

### 3.4. Consistency Optimization Framework

**Figure 9:**
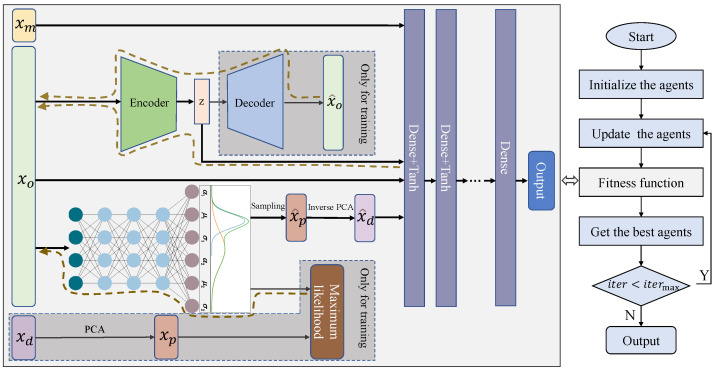
The proposed consistency optimization framework.

The final optimization framework of the method is shown in Figure 9. The right side of the figure shows the general population-based optimization algorithm flow. The core of the method is to construct a suitable fitness function for the optimizer. It can be seen that the final prediction model denoted as SERCAM integrates the aforementioned distribution prediction model as well as the operational variable self-supervised feature extraction model. Theraw materials grade parameters, operating parameters, self-coded extracted features, and sampled data from the detection variables corresponding feature distributions are integrated and input into the dense layers to achieve production index prediction. During the model training phase, the sampled data are replaced by the practical detection variables data. It is important to note that the detection variable data are only used in the model training phase.

In the figure, $x_{m}$, $x_{o}$, $x_{d}$ represent the materials grade parameters, operational parameters, and detection variables, respectively. $x_{p}$ represents the feature variables after decoupling the detection variables. ${\hat{x}}_{p}$, ${\hat{x}}_{d}$ denote the corresponding sampling data in the prediction stage, respectively. ${\hat{x}}_{o}$ represents the reconstructed operational parameters sample. The dashed boxes in the figure are used only for the model training phase. The brown dashed line with arrows indicates the backpropagation links.

## 4. Experiments and Discussion

To verify the validity, the method was validated on a empirical production data set. The data are the 2021 production data from the No. 2 blast furnace of a steel group’s ironmaking plant in a southwestern province of China. This ironmaking plant is different from normal ironmaking plants with stable and high-grade raw materials. They expanded many iron ore sources to reduce raw material costs, leading to low and fluctuating iron ore grades. Therefore, raw material information needs to be used as input features when forecasting production indexes. When the ingredients’ grades do not fluctuate much, they can usually be considered as stable system information instead of being used as input variables. The number of input variables affects the complexity and information extraction capability of the model. Sinter and coke, as the most prominent raw materials for the studied blast furnace, have fluctuating grades. Their grade information will be used as input to the model.

Pig iron is the product of the blast furnace production process, and the phosphorus content of pig iron is one of the important indicators of the quality of pig iron. As a harmful element, phosphorus directly affects the efficiency of smelting and production costs in the subsequent steelmaking process. Therefore, the optimization of the phosphorus content of the pig iron is of great importance. It should be noted that, for ore materials with low phosphorus content, it is difficult for the blast furnace to reduce the phosphorus content of the iron. For ores with high phosphorus content, proper operation of the blast furnace can reduce the phosphorus content in the iron. The optimization of phosphorus content is of considerable significance in the low and fluctuating ore grades of this ironmaking plant.

In order to optimize the phosphorus content of iron, the variables related to the phosphorus content of iron need to be determined first. The required variables were identified by correlation analysis and expert experience, as shown in Table 1, including ore grade parameters, operation parameters, and detection variables [25].

As mentioned before, the top temperature and pressure are considered uncontrollable variables. The simulation test is carried out based on the data set containing 10,000 blast furnace process samples. The variables are normalized to facilitate model training.

**Figure 10:**
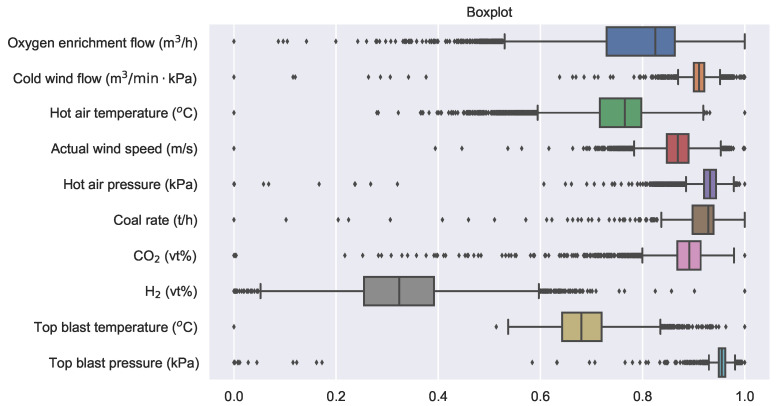
Box-plot for related variables (extreme outlier).

There are always some anomalous samples, as shown in Figure 10. The horizontal axis of the figure represents the normalized variable values, while the vertical axis represents the variables. The left end of the solid line represents the minimum value of the variable, the right end represents the maximum value, and the rectangle covers the values from the lower quartile to the upper quartile. The black dots symbolize outliers. Box plots are used to identify and eliminate outliers. Outliers were not removed directly but replaced with the mean of the normal samples before and after them. The data set is divided at a ratio of 8:1:1 into a training set with 8000 samples, a validation set, and a test set both with 1000 samples.

To verify the effectiveness of the established MDN model in predicting the posterior distribution of uncontrollable variables under the given operational parameters, the method was tested on the above-mentioned data set. In addition, to measure the difference between the predicted and true distributions, the CRPS index is introduced:

(8) $$CRPS=\frac{1}{N}\sum_{i=1}^{N}{\int_{-\infty }^{+\infty }\left[CDF_{i}\left(t\right)-H\left(t-y_{i}\right)\right]}^{2}dt$$

where $CDF_{i}$ denotes the posterior cumulative distribution function for the *i*-th sample, *H* denotes the Heavyside step function and $y_{i}$ denotes the observation. According to the definition, CRPS is zero when the predicted distribution is exactly the same as the true distribution. CRPS increases when the predicted distribution is overly concentrated, dispersed, or deviates too far from the observed value.

**Figure 11:**
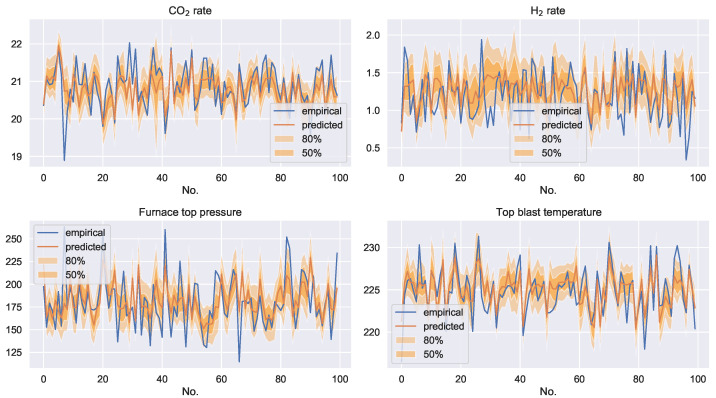
Prediction results of the posterior distribution of uncontrollable variables. The horizontal axis of each subplot indicates the numbers of the samples. The vertical axis indicates the value of each variable.

The plots of the prediction results of the MDN model for several uncontrollable variables are shown in Figure 11. The blue line in the graph represents the empirical data, the orange line represents the mean of the predicted distribution, and the orange shading represents the predicted distribution interval.

It can be seen that the model is able to determine the uncontrollable variables in a narrow distribution interval for given operating variables, and the size of the distribution varies with the operating parameters.

The number of Gaussian components as an essential hyperparameter of the MDN model has an important impact on the model prediction performance. To study the effect, the models are tested under different Gaussian component numbers.

Table 2 shows the CRPS of the prediction results of the model for the features decoupled from PCA at different Gaussian component numbers. Considering the model prediction accuracy as well as the model complexity, subsequent experiments set the number of Gaussian components as 2.

**Figure 12:**
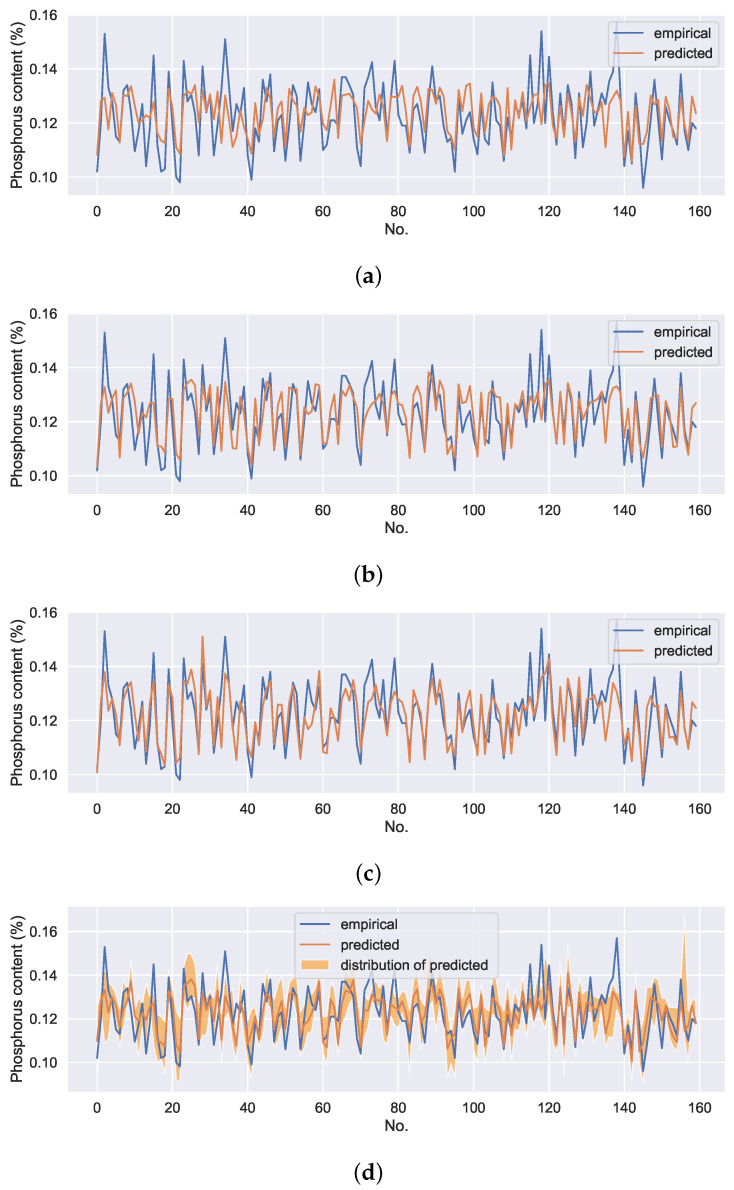
The prediction results of different models under different input conditions. (**a**) the prediction results of DNN without UV; (**b**) the prediction results of DNN with UV; (**c**) the prediction results of βSER; (**d**) the prediction results of SERCAM.

To verify the effectiveness of the proposed method, four experiments were conducted. The first experiment was performed on a DNN model with ore grade parameters, operating parameters as inputs, and phosphorus content of pig iron as output. It can be represented as DNN without uncontrollable variables (UV). The predicted results are shown in the first row in Figure 12. The second experiment was also performed on a DNN model with ore grade parameters, operating parameters, and detection variables as inputs and phosphorus content of pig iron as output. It can be represented as DNN with UV. The predicted results are shown in the second row in Figure 12. The third experiment was performed on a βSER model with ore grade parameters, operating parameters, and detection variables as inputs and phosphorus content of pig iron as output. According to the experimental results, the key hyperparameters of the model, deep feature dimension, and balance coefficient β were set as 5 and 0.8, respectively. The predicted results are shown in the third row in Figure 12. The final experiment was performed on SERCAM with ore grade parameters, operating parameters as inputs, and phosphorus content of pig iron as output. The hyperparameters of the self-encoding feature extraction module are consistent with the above βSER model. The number of samples of features corresponding to the uncontrollable variables in the CAM module is set as 50. The predicted results are shown in the last row in Figure 12. Each data point on the orange line in the plot is the mean of the 50 predicted values. The orange shading denotes the distribution of the predictions. For the sake of observation, only 160 data on the test set are presented. RMSE of the models on the entire test set are shown in Table 3. The hyperpameters of the models are shown in Table 4.

It can be seen that βSER can significantly improve the model prediction accuracy compared with the general DNN model. Since the uncontrollable variables are not included as inputs, the SERCAM model still has a slight disadvantage in prediction accuracy compared to the models with the input of detection variables. However, SERCAM not only has higher prediction accuracy compared to the DNN model without uncontrollable variables inputs but also is capable of giving uncertainty estimates of the predicted values while ensuring the controllability of the model inputs.

**Figure 13:**
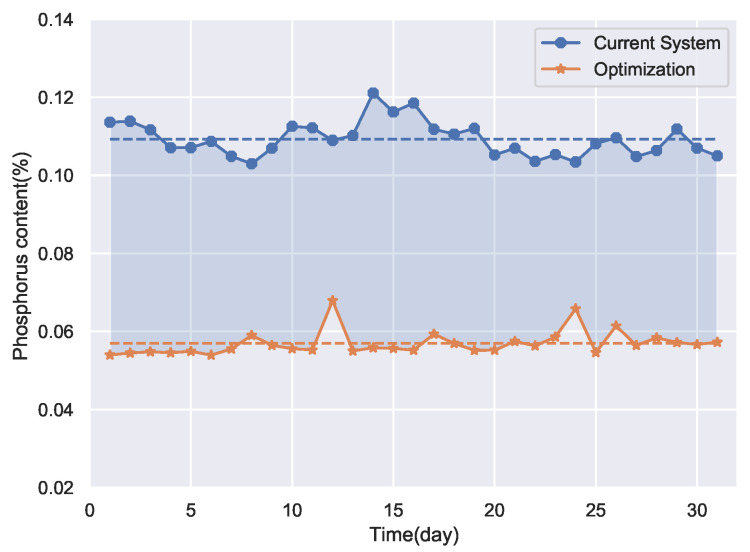
Optimization results of the conventional method.

After the establishment of the prediction models, the optimization of the production process can be carried out. Firstly, the phosphorus content of the pig iron is optimized with conventional optimization method that ignores the uncontrollability of detection variables. Take the DNN with the UV model as the fitness function of the Gray Wolf optimizer to perform process optimization. The optimization method was simulated on the production data of the ironmaking plant for 31 days in December 2021. The optimization results are shown in Figure 13.

**Figure 14:**
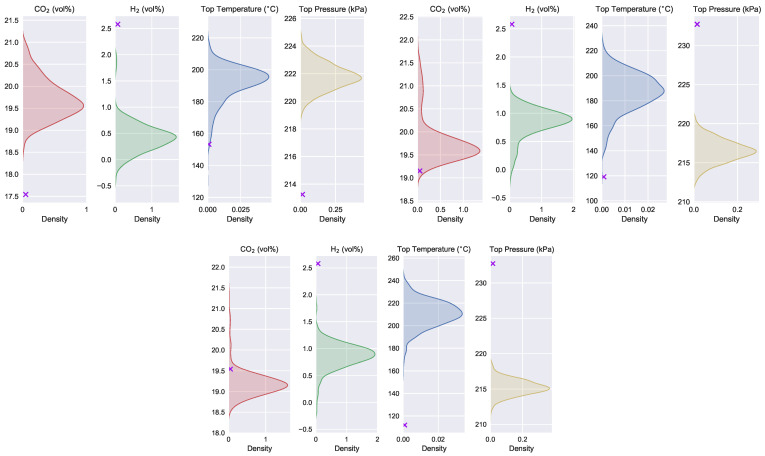
Inconsistency of operating parameters and detection variables recommended by the conventional optimization method.

The shaded area in Figure 14 represents the distribution of the uncontrollable variables consistent with the recommended operating parameters given by the optimization method for the ore grade of the first three days of December. The symbols of the crosses in the figure indicate the values of the recommended uncontrollable variables given by the optimization algorithm.

From the optimization results, it seems that the method has achieved excellent effects. However, it can be seen from Figure 14 that the recommended operating parameters and uncontrollable variables are not consistent. The recommended uncontrollable variables fall on the edge or even outside of the distribution that is consistent with the recommended operating parameters. This means that the optimization results shown in Figure 13 have little or not even a probability of occurring.

**Figure 15:**
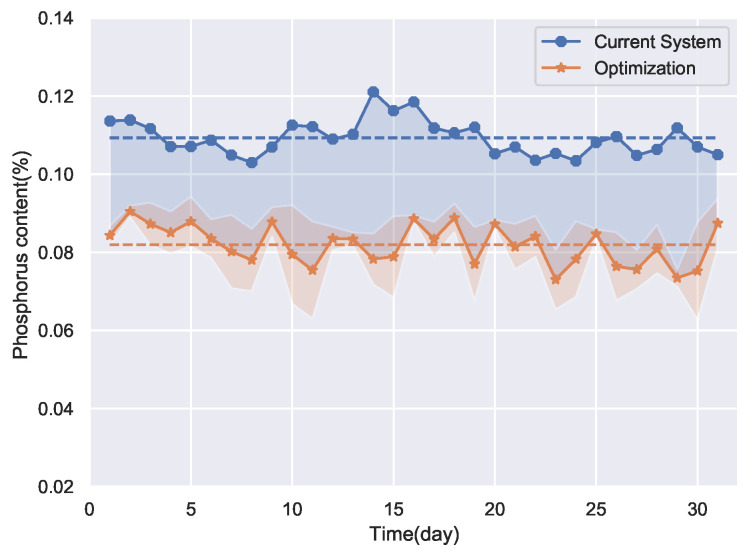
Optimization results of the proposed method.

The optimization results of the proposed method are shown in Figure 15. The SERCAM model is used as the fitness function of the multi-objective gray wolf optimizer, and the optimization is performed to find the smallest possible mean and standard deviation of the phosphorus content of the pig iron.

**Figure 16:**
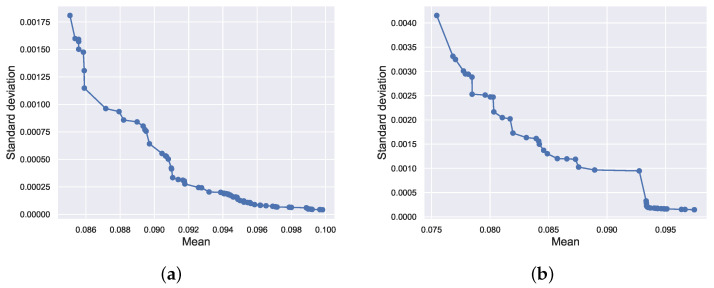
(**a**) The Pareto front solutions for 4 December given by the proposed method; (**b**) the Pareto front solutions for 11 December.

Figure 16a,b are the Pareto frontier solutions obtained by optimization for 4 and 11 December, respectively. The optimal solutions in Figure 15 are solutions with the smallest mean among the frontier solutions. The pink shading in Figure 15 indicates the fluctuation range of the optimization results. It can be seen that the proposed optimization method is not only able to obtain the expected value of the optimization result but also the uncertainty of the optimization solution. It makes the optimization method more reliable and valid than the conventional methods.

## 5. Conclusions

In some industrial processes, information about uncontrollable variables is the key to ensuring the accuracy of soft sensor models. In this paper, the unreliability of the conventional data-driven meta-heuristic method for blast furnace production process optimization is pointed out because it requires detection variables to assist in establishing a more accurate fitness function, while the detection variables are not controllable. A consistency optimization framework based on a controllability-assured soft sensor modeling method, which improves the prediction accuracy by extracting the information of uncontrollable variables while ensuring the controllability of model inputs, is proposed. Under the condition that uncontrollable variables are not used as input, the proposed method improves the prediction accuracy of the model by extracting relevant information and realizes the uncertainty estimation of the prediction target. The method transforms the original problem into a multi-objective optimization problem so that it has the ability to give both the optimization solution and the reliability evaluation of the solution, and finally achieves the reliable optimization of the blast furnace process. Then, the model was tested on the real data set. In order to verify the performance of the proposed model, prediction experiments were carried out under various input conditions and models. The results indicated that our model achieved an outstanding performance. Moreover, optimization experiments are also conducted to analyze the proposed method. The results also point out that the optimized solution of our method is more reliable.

Uncertainty estimation is of great significance for the application of algorithms. Simple theoretical results in a harsh industrial production environment can bring great uncertainty to production. Only a reliable estimation of the uncertainty of the method can make the method truly applicable. Therefore, in future works, we will focus on uncertainty estimation of data-based modeling and optimization methods.

## Figures and Tables

**Table 1:** Related variables.

Variables	Unit	Variable Type	Controllable
Cold wind flow	m${}^{3}$/min·kPa	Operation Variable	✓
Pulverized coal injection	t/h	Operation Variable	✓
Wind speed	m/s	Operation Variable	✓
Oxygen enrichment flow	m${}^{3}$/h	Operation Variable	✓
Hot air temperature	°C	Operation Variable	✓
Hot wind pressure	kPa	Operation Variable	✓
Top blast pressure	kPa	Detection variable	×
Top temperature	°C	Detection variable	×
CO${}_{2}$ rate	vt%	Detection variable	×
H${}_{2}$ rate	vt%	Detection variable	×
SiO${}_{2}$ content	wt%	Sinter grade	×
CaO content	wt%	Sinter grade	×
TFe content	wt%	Sinter grade	×
M40	%	Coke grade	×
CRI	%	Coke grade	×
CSR	%	Coke grade	×

**Table 2:** CRPS values of the model with different Gaussian component numbers.

Numbers	Feature 1	Feature 2	Feature 3	Feature 4
1	0.0655	0.0602	0.0461	0.0710
2	0.0639	0.0578	0.0442	0.0698
3	0.0664	0.0601	0.0452	0.0715
4	0.0657	0.0592	0.0473	0.0717

**Table 3:** RMSE of the models.

	DNN without UV	DNN with UV	βSER	SERCAM
RMSE	0.0099	0.0084	0.0074	0.0089

**Table 4:** Hyperpameters of the methods.

Models	Hyperpameters					
DNN without UV	Learning rate			Hidden layers		
	0.001			64-128-32		
DNN with UV	Learning rate			Hidden layers		
	0.001			64-128-32		
βSER	Learning rate	β	Encoder hidden layers	Encoder feature dimension	Decoder hidden layers	Dense layers
	0.001	0.8	64-128-64	5	64-128-64	64-128-64
SERCAM	βSER module	CAM module				
		Learning rate	Hidden layers	Gaussian components Num	Sampling Num	
	Same as above	0.001	128-32	2	50	

## Data Availability

Data sharing not applicable.
